# A simple model for vacancy order and disorder in defective half-Heusler systems

**DOI:** 10.1107/S2052252520005977

**Published:** 2020-06-06

**Authors:** Nikolaj Roth, Tiejun Zhu, Bo B. Iversen

**Affiliations:** a Center for Materials Crystallography, Department of Chemistry, Aarhus University, Aarhus 8000, Denmark; b State Key Laboratory of Silicon Materials, School of Materials Science and Engineering, Zhejiang University, Hangzhou 310027, People’s Republic of China

**Keywords:** defective half-Heuslers, diffuse scattering, short-range order, correlated disorder, thermoelectrics, inorganic materials, materials modeling, properties of solids

## Abstract

Defective half-Heusler systems *X*_1−*x*_ *YZ* with large amounts of intrinsic vacancies, such as Nb_1−*x*_CoSb, Ti_1−*x*_NiSb and V_1−*x*_CoSb, are a group of promising thermoelectric materials. Even with high vacancy concentrations they maintain the average half-Heusler crystal structure. These systems show high electrical conductivity but low thermal conductivity arising from an ordered *YZ* substructure, which conducts electrons, while the large amounts of vacancies in the *X* substructure effectively scatters phonons. Using electron scattering, it was recently observed that, in addition to Bragg diffraction from the average cubic half-Heusler structure, some of these samples show broad diffuse scattering indicating short-range vacancy order, while other samples show sharp additional peaks indicating long-range vacancy ordering. Here it is shown that both the short- and long-range ordering can be explained using the same simple model, which assumes that vacancies in the *X* substructure avoid each other. The samples showing long-range vacancy order are in agreement with the predicted ground state of the model, while short-range order samples are quenched high-temperature states of the system. A previous study showed that changes in sample stoichiometry affect whether the short- or long-range vacancy structure is obtained, but the present model suggests that thermal treatment of samples should allow controlling the degree of vacancy order, and thereby the thermal conductivity, without changes in composition. This is important as the composition also dictates the amount of electrical carriers. Independent control of electrical carrier concentration and degree of vacancy order should allow further improvements in the thermoelectric properties of these systems.

## Abstract 2

Defective half-Heuslers, *X*_1−*x*_ *YZ*, with different degrees of short- and long-range vacancy order can be explained using a simple model. In this model, the vacancies avoid being nearest or next-nearest neighbors. The model reproduces previously observed diffuse electron-diffraction data. The findings suggest a method for further improving thermoelectric efficiency of these materials.

## 1. Introduction   

To combat climate change while supplying a growing world population with affordable and clean energy, improvements in sustainable energy production are needed (UN General Assembly, 2015 ▸). One of several technologies that are useful to obtain such a goal is thermoelectric energy conversion, which allows direct conversion of waste heat into electrical energy (He & Tritt, 2017 ▸). To do so efficiently, thermoelectric materials must have a high electrical conductivity together with a low thermal conductivity (Snyder & Toberer, 2008 ▸). High electrical conductivities are usually found in highly ordered crystalline materials, while low thermal conductivities are usually associated with disordered or amorphous materials. One way to overcome this apparent paradox is through materials that have a long-range crystalline-ordered substructure with a disordered substructure. The ordered substructure should be responsible for the conduction of electrons while the disordered substructure will efficiently scatter phonons. Such materials are typically understood using the phonon-glass electron crystal concept (Slack, 1995 ▸; Snyder & Toberer, 2008 ▸). Examples include clathrates and skutterudites, where conducting crystalline-network structures have additional guest atoms in loosely bonded positions, typically large voids. This allows the guest atoms to ‘rattle’ independently of the network and thereby scatter phonons efficiently. Another example is Cu_2−*x*_Se, which consists of a long-range ordered Se substructure allowing electronic transport, while the Cu substructure is disordered. At room temperature, the Cu atoms are ordered in two dimensions but disordered along the third dimension, and above ∼400 K the Cu substructure becomes almost liquid like (Liu *et al.*, 2012 ▸; Roth & Iversen, 2019 ▸). To further understand such phonon-glass electron crystal materials, accurate knowledge of their atomic structure is required.

Ordered crystalline materials give rise to sharp Bragg peaks in scattering experiments. Conventionally, the structures of crystalline materials are solved by analyzing the Bragg peak intensities from either X-ray or neutron-scattering experiments. The sharp Bragg peaks contain information about the unit-cell average structure. For perfectly ordered crystals, this is the same as the complete structure. However, for crystals containing disorder this is no longer the case. For crystals with large movements of atoms, such as clathrates, the average structure only shows the smeared-out average electron density of the moving atoms. For crystals with substitutional disorder, only the average electron density of a site can be known from analysis of Bragg intensities, and all information about local correlations is lost in the average structure.

To obtain information about the local correlations it is necessary to analyze the weak diffuse scattering, which contains information about disorder and short-range order (Billinge & Egami, 2003 ▸; Keen & Goodwin, 2015 ▸; Welberry, 2004 ▸; Welberry & Weber, 2016 ▸). In crystals with correlated movements of atoms, the diffuse scattering can show the actual instantaneous configuration of atoms. A recent example relevant to thermoelectric research is the existence of local dynamic dipole formation in PbTe (Sangiorgio *et al.*, 2018 ▸). In crystals with substitutional or vacancy disorder, the diffuse scattering contains information about the short-range order structure. As an example, this was recently used to show that thermoelectric Cu_2−*x*_Se at room temperature contains ordered two-dimensional layers of Cu, whereas the structure is disordered along the third dimension (Roth & Iversen, 2019 ▸).

Half-Heusler compounds with general composition *XYZ*, such as ZrNiSn, ZrCoSb and NbFeSb, among others, have been intensively studied for use as thermoelectric materials because of their favorable properties (Zhu *et al.*, 2015 ▸; He *et al.*, 2017 ▸; Zeier *et al.*, 2016 ▸; Fu *et al.*, 2012 ▸, 2015 ▸; Nolas *et al.*, 2006 ▸; Sakurada & Shutoh, 2005 ▸; Yan *et al.*, 2011 ▸; Shen *et al.*, 2001 ▸). These compounds all have a valence electron count of 18, which for the half-Heusler structure results in the filling of all bonding electronic states, giving a highly stable structure. As these compounds have all their states filled up to the band gap, they behave as semiconductors. The main drawback of these compounds has been their relatively high lattice thermal conductivities, which result from their simple crystal structure, although nanostructuring and alloying have been applied to reduce this issue (Graf *et al.*, 2011 ▸; Zhu *et al.*, 2017 ▸). Recently a new group of nominal 19-electron half-Heusler compounds have been studied, *e.g.* VCoSb, NbCoSb and TiNiSb (Huang *et al.*, 2015 ▸; Zhang *et al.*, 2016 ▸; Zeier *et al.*, 2017 ▸; Xia *et al.*, 2018 ▸, 2019 ▸; Anand *et al.*, 2018 ▸). With the extra electron compared with 18-electron half-Heusler compounds, these new compounds should be expected to be metallic and less stable. However, instead they have been found to behave as strongly doped semiconductors with much lower thermal conductivities than the 18-electron systems (Huang *et al.*, 2015 ▸; Xia *et al.*, 2018 ▸, 2019 ▸; Zeier *et al.*, 2017 ▸; Zhang *et al.*, 2016 ▸), making them very suitable for thermoelectric applications. The explanation for this is that the systems in fact have large deficiencies of the *X* element, getting them closer to an 18-electron composition. As an example the nominal 19-electron NbCoSb was shown to consist of a phase with a composition close to the 18-electron Nb_0.8_CoSb and additional Nb-rich impurity phases. The Nb_1−*x*_CoSb phase has been reported with stoichiometries Nb_0.84_CoSb by Zeier *et al.* and Nb_0.79_CoSb—Nb_0.83_CoSb by Xia *et al.* (Xia *et al.*, 2018 ▸; Zeier *et al.*, 2017 ▸).

These new compounds are therefore better described as defective half-Heusler *X*_1−*x*_ *YZ* systems. Interestingly, these compounds still maintain the average cubic half-Heusler crystal structure, even though they often have ∼20% deficiency of the *X* element (Xia *et al.*, 2018 ▸; Zeier *et al.*, 2017 ▸). Their electronic structure can be explained using the 18-electron rule, where the additional/fewer Nb atoms compared with Nb_0.8_CoSb will act as heavy dopants (Zeier *et al.*, 2017 ▸).

Because of impurities formed during synthesis, additional characterization of these compounds is needed to know the actual stoichiometry of the half-Heusler phases. The sample with nominal composition NbCoSb produced by Zeier *et al.* was found to have a composition of about Nb_0.84_CoSb using Rietveld refinement of powder X-ray diffraction data (Zeier *et al.*, 2017 ▸). Using an electron-probe micro-analyzer wavelength-dispersive spectroscope, Xia *et al.* measured the stoichiometries of the half-Heusler phases in their samples (Xia *et al.*, 2018 ▸). For all phases with nominal stoichiometry of Nb_1−*x*_CoSb in the range *x* = 0–0.25 they found that the stoichiometry of the half-Heusler phase only varies in the interval *x* = 0.17–0.21. For nominal compositions outside this region, additional impurity phases were observed.

**Figure 1:**
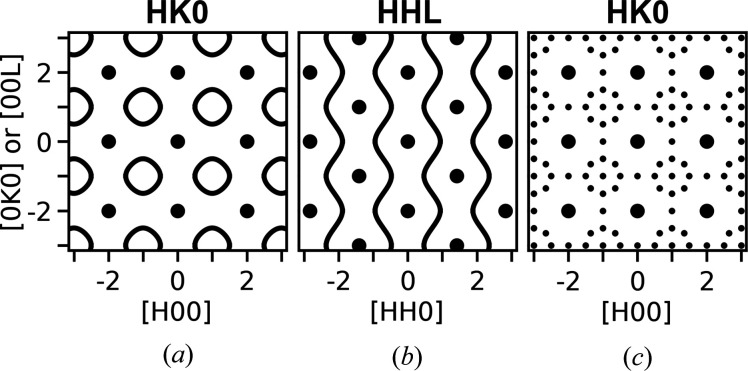
Sketches showing the scattering measured for a number of defective half-Heusler systems by Xia *et al.* (2019 ▸). (*a*), (*b*) Sketches that show the *HK*0 and *HHL* planes for short-range order systems Nb_0.8_CoSb, Ti_0.9_NiSb, V_0.9_CoSb and Nb_0.8_Co_0.92_Ni_0.08_Sb. (*c*) A sketch showing the *HK*0 plane for ordered Nb_0.82_CoSb and Nb_0.84_CoSb. Large dots indicate the reflections from the average structure, lines indicate diffuse scattering and small dots indicate weak additional peaks.

With this large number of missing Nb atoms, the question arises of how these vacancies are distributed. Zeier *et al.* suggested an ordered tetragonal vacancy structure for Nb_0.8_CoSb based on density-functional theory (DFT) calculations (Zeier *et al.*, 2017 ▸). Recently, Xia *et al.* investigated the electron scattering from several of these defective half-Heusler systems (Xia *et al.*, 2019 ▸). For Nb_0.8_CoSb, Ti_0.9_NiSb, V_0.9_CoSb and Nb_0.8_Co_0.92_Ni_0.08_Sb, similar scattering patterns were observed showing both strong Bragg peaks from the average cubic half-Heusler structure and weak diffuse scattering coming from short-range order of vacancies. In the *H* *K*0 plane of the scattering, diffuse rings were found around the systematically extinct reflections with both odd *H* and *K*. This is sketched in Fig. 1 ▸(*a*). In the *HHL* plane (spanned by *HH*0 and 00*L*) continuous waves of diffuse scattering were observed between rows of Bragg peaks along the 00*L* direction, as sketched in Fig. 1 ▸(*b*). In the Nb_1−*x*_CoSb system, Xia *et al.* also observed several ordered structures depending on the nominal stoichiometry of the samples (Xia *et al.*, 2019 ▸). For the Nb_0.81_CoSb sample, the scattering in the *HK*0 plane showed two additional peaks on the diffuse scattering ring on opposite sides of the ring along one of the [*H*00] directions, and further peaks were found halfway between the main reflections of the structure in the same direction.

For the samples with nominal stoichiometry Nb_0.82_CoSb and Nb_0.84_CoSb [measured stoichiometry Nb_0.81_CoSb and Nb_0.82_CoSb (Xia *et al.*, 2018 ▸)] the circles of diffuse scattering were mainly gone and replaced by an 8-point ring, with additional very weak peaks in the center of the ring and between main reflections along all [100] directions, sketched in Fig. 1 ▸(*c*).

As very similar types of short-range order diffuse scattering have been observed in several different half-Heusler systems it seems to be a common feature of these systems. Here we give a simple model for both the diffuse scattering as well as the ordered structures observed by electron diffraction by Xia *et al.* (2019) ▸. The model only considers the positions of vacancies on the *X* substructure of the structure.

## 2. Theory   

**Figure 2:**
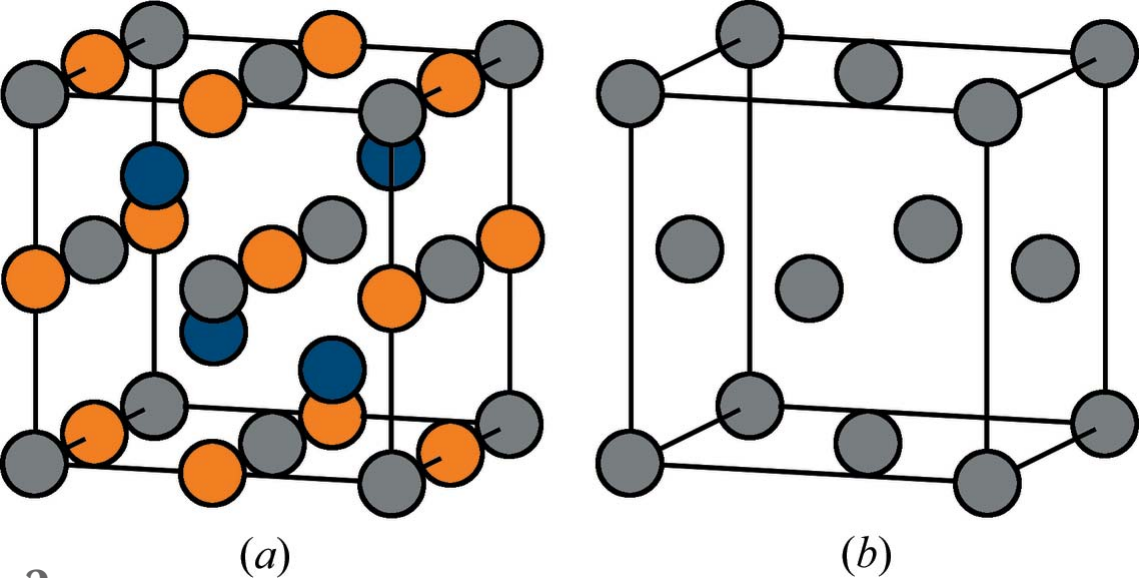
Crystal structure of the defective *X*_1−*x*_ *YZ* half-Heusler systems. (*a*) The full structure with *X* (gray), *Y* (blue) and *Z* (orange). (*b*) The *X*/vacancy f.c.c. substructure.

The average structure of the defective *X*_1−*x*_ *YZ* half-Heusler systems is shown in Fig. 2 ▸(*a*). The half-Heusler structure has *X* on a face-centered cubic (f.c.c.) lattice with *Z* in the octahedral positions and *Y* in half of the tetrahedral positions. *X* and *Y* together form the sphalerite structure while *X* and *Z* together form the rock-salt structure. As these systems have a deficiency of *X*, there will be vacancies in the structure. It was found that there are no apparent anti-site defects in these systems (Zeier *et al.*, 2017 ▸) as there are for other half-Heusler systems (Larson *et al.*, 2000 ▸; Kirievsky *et al.*, 2013 ▸). The vacancies are therefore limited to the f.c.c. substructure of the half-Heusler structure, as shown in Fig. 2 ▸(*b*).

**Figure 3:**
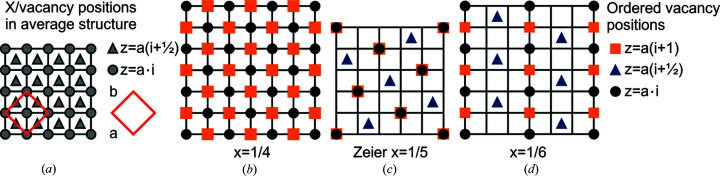
Vacancy positions on the f.c.c. lattice as seen from above. (*a*) The possible vacancy positions. Gray circles mark sites with integer *z* coordinates and gray triangles mark sites with half-integer *z* coordinates. The *a* and *b* axis of the unit cell are along the diagonal as shown by the red square. (*b*) One possible ordering of vacancies for *x* = 1/4. In this ordering there are no vacancies with half-integer *z* coordinates. This ordering has no nearest-neighbor vacancies, and each vacancy has four next-nearest neighbor vacancies. (*c*) The vacancy ordering with *x* = 1/5 suggested by Zeier *et al.* (2017 ▸) giving a tetragonal structure. Note that every second layer is identical. This ordering has no nearest-neighbor vacancies, but every vacancy has two next-nearest neighbor vacancies. (*d*) One of several possible vacancy orderings for *x* = 1/6 where there are no nearest and no next-nearest neighbor vacancies. Other possible orderings are shown in the Supporting information.

The question of the atomic short- and long-range order can therefore be simplified to how the vacancies are distributed on the f.c.c. lattice. To illustrate different vacancy distributions we will use a different representation of this structure, where the f.c.c. substructure is viewed from above, as shown in Fig. 3 ▸(*a*). Here the gray circles show the sites with whole integer *z* coordinates and the gray triangles show the sites with half-integer *z* coordinates. In this view, the unit-cell axes are along the diagonal, as marked by the red square. This representation is convenient for representing the ordered vacancy structures as shown below.

We make the simple assumption that vacancies avoid each other, and only take into account nearest and next-nearest neighbor interactions. When there are more than 1/4 vacancies in the *X* substructure it is not possible to arrange the vacancies such that there are no nearest neighbors. When *x* = 1/4 it is possible to make an ordered arrangement with no nearest neighbors, but with each vacancy having four out of six next-nearest neighbors also vacant. This arrangement is illustrated in Fig. 3 ▸(*b*). In order to both completely avoid nearest and next-nearest neighbor vacancies, 1/6 sites need to be vacant. Several possible arrangements will satisfy these conditions, and one such arrangement is shown in Fig. 3 ▸(*d*). More are shown in the Supporting information. This means that for *x* < 1/4 nearest neighbors can be eliminated and for *x* < 1/6 also all next-nearest neighbors can be eliminated. In cases where 1/4 > *x* > 1/6 there has to be some next-nearest neighbor vacancies if nearest-neighbor vacancies are still to be avoided.

The ordered vacancy structure for *x* = 1/5 suggested by Zeier *et al.* (2017 ▸) is illustrated in Fig. 3 ▸(*c*). In this structure, each vacancy has no nearest neighbors but two next-nearest neighbors. However, if instead of adapting the ordered *x* = 1/5 structure the vacancy substructure creates domains of *x* = 1/6 and *x* = 1/4 type structures there will be a lower average number of next-nearest neighbors. This will allow 3/5 of the vacancies to go into the *x* = 1/6 structure, leaving 2/5 of the vacancies in the *x* = 1/4 structure. This will give an average number of next-nearest neighbors per vacancy of 1.6, somewhat lower than in the structure suggested by Zeier *et al.* (2017 ▸). This would suggest that domain-phase separation of the substructure is more favorable for 1/4 > *x* > 1/6.

For *x* < 1/6 there are many states all obeying no nearest and next-nearest neighbor vacancies and the model system will be disordered. To further investigate the vacancy distribution. we can simulate the system using Monte Carlo simulations for different compositions, *x* and temperatures. As we are only considering nearest and next-nearest neighbor interactions of the vacancies, the energy of the model system can be written as a sum over all *N*_vac_ vacancies,
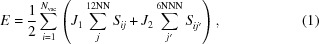
where the sum over *j* covers the 12 nearest-neighbor (NN) sites of vacancy *i*. *S*_*ij*_ is 1 if site *j* has a vacancy and 0 if it is occupied. Similarly, the sum over *j*′ is over the six next-nearest neighbors (NNN) of vacancy *i*, where *S*_*ij*′_ is 1 if site *j*′ is vacant and 0 if it is occupied. In this formalism, an energy of *J*_1_ is assigned to a nearest-neighbor vacancy pair and *J*_2_ for a next-nearest neighbor vacancy pair.

## 3. Methods   

Monte Carlo simulations were carried out using a custom code written in *Python*. A box of 24 × 24 × 48 sites with the appropriate number of vacancies was started in either a random configuration or from one of the ordered states. In cases where the ordered starting state was used for a different *x* than that of the ordered state, either additional vacancies were added randomly or random vacancies were removed in order to have the correct number of total vacancies. Vacancies were allowed to move to nearest-neighbor positions following the Metropolis algorithm (Metropolis *et al.*, 1953 ▸) with the energy expression given in equation (1). In cases where convergence was hard to reach, a simulated annealing approach was used, where the temperature of the simulation was continuously lowered. Each simulation was repeated 20 times to increase the statistics of the following scattering calculation. The simulations shown in this article used *J*_2_/*J*_1_ = 0.48 as the relative value for the next-nearest neighbor energy. This value was chosen because a *J*_2_/*J*_1_ lower than 0.5 reproduces the observed ground-state scattering well while 0.45 or higher reproduces the observed short-range order scattering at higher simulated temperatures well. See the Supporting information for simulations with other values of *J*_2_/*J*_1_.

The scattering patterns of the configurations were calculated using the software *Scatty* (Paddison, 2019 ▸) with Nb on the positions of the occupied *X* sites. X-ray scattering factors were used.

## 4. Results   

**Figure 4:**
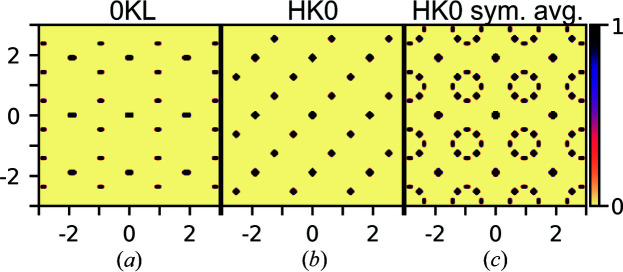
Calculated scattering from the ordered *x* = 1/6 structure. (*a*), (*b*) The 0*KL* and *HK*0 planes which are equivalent in the average cubic structure. (*c*) The *HK*0 plane of the scattering symmetry averaged to the average structure, which would be expected for micro-domains with different but equivalent orientations.

As the average structure is cubic, while the proposed ordered vacancy structures are not, different scattering signals should be observed in directions which are equivalent in the average cubic structure. As an example, the calculated scattering from the ordered *x* = 1/6 model is shown in Figs. 4 ▸(*a*) and 4(*b*) for the 0*KL* and *HK*0 planes, which are symmetry equivalent in the average cubic structure. The 0*KL* plane shows additional peaks corresponding to those Xia *et al.* observed for their Nb_0.81_CoSb sample (Xia *et al.*, 2019 ▸), although without the additional diffuse scattering they observed. The *HK*0 plane is different with the additional peaks forming rows along one diagonal. The *H*0*L* plane, not shown here, is equivalent to the 0*KL* plane. It could be expected that this type of system will have many micro-domains, where different regions of the sample will orient along the different equivalent directions of the average cubic structure. If the scattering of the ordered *x* = 1/6 structure is symmetrized to the Laue symmetry of the average structure, the scattering pattern shown in Fig. 4 ▸(*c*) is obtained. Now the 8-point rings have emerged, which are very similar those observed in several samples by Xia *et al.* (2019 ▸) but without the peaks at the center of the ring. This however shows that most of the reported samples have micro-domain structure with different orientations, which are symmetry equivalent in the average cubic structure. The eight points in the observed rings do therefore not all come from one domain, which would require an incommensurate modulated structure, but come in pairs from different orientations of a commensurate superstructure. In the following, we will focus on the cubic symmetry averaged scattering of the different phases, as these are the patterns most likely to be observed in an experiment where the sample will contain many micro-domains.

**Figure 5:**
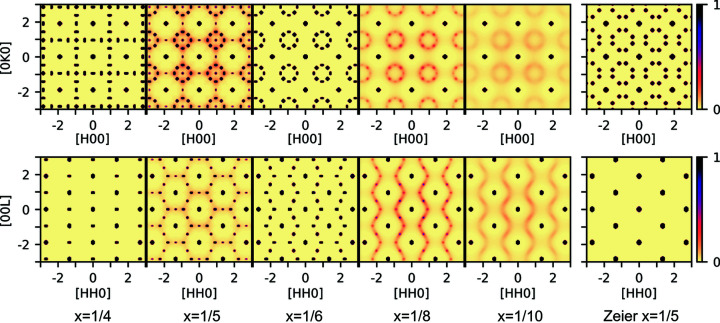
Calculated scattering for the ground states at different compositions in the *HK*0 (upper row) and *HHL* planes (lower row). For all these models the scattering has been averaged to the symmetry of the average structure to simulate the expected measurement for a sample with multiple domains. The scattering for *x* = 1/4, *x* = 1/6 as well as the *x* = 1/5 structure suggested by Zeier *et al.* (2017 ▸) are calculated from the ordered vacancy structures shown in Fig. 3 ▸. For the *x* = 1/5, *x* = 1/8 and *x* = 1/10 systems the scattering is calculated from the Monte Carlo simulated structures. The calculated scattering for several other possible ground-state vacancy structures and the scattering in non-symmetry averaged planes are shown in the Supporting information.

Fig. 5 ▸ shows the scattering patterns for the ground-state structures for different compositions. The figure shows both the *HK*0 (upper row) and *HHL* planes (lower row) which have been symmetry averaged to the Laue symmetry of the average cubic structure. The scattering from the ordered *x* = 1/4 structure presented in Fig. 3 ▸(*b*) is shown in the first column of Fig. 5 ▸. Characteristic of the *HK*0 plane are the rows of additional peaks along odd-integer *H* and *K*. The scattering of the ordered *x* = 1/6 structure presented in Fig. 3 ▸(*d*) is shown in the third column of Fig. 5 ▸. As was shown before, the *HK*0 plane has the 8-point ring without a peak at the center. In the region 1/4 > *x* > 1/6, the structure obtained from Monte Carlo simulation has scattering which has both features from the *x* = 1/4 and *x* = 1/6 structures with additional diffuse scattering. This is shown in the second column of Fig. 5 ▸ for a simulation with *x* = 1/5. The 8-point rings here also have peaks at their center and there are additional peaks between the main reflections. In this model there are inclusions of the *x* = 1/4 type regions in the *x* = 1/6 type structure. The scattering of this model matches that reported by Xia *et al.* for samples with nominal stoichiometry Nb_0.82_CoSb and Nb_0.84_CoSb [measured stoichiometry Nb_0.81_CoSb and Nb_0.82_CoSb (Xia *et al.*, 2018 ▸)], as sketched in Fig. 1 ▸(*c*). The structure suggested by Zeier *et al.* for *x* = 1/5 based on DFT calculations (Zeier *et al.*, 2017 ▸) has a different scattering pattern to what has been observed, as seen in the last column of Fig. 5 ▸. As suggested in the theoretical section[Sec sec2] of this article, it might be more favorable to have separation into domains of the ordered *x* = 1/4 and *x* = 1/6 types than to adapt the structure suggested by Zeier *et al.* (2017 ▸), and indeed the scattering from the ordered structures measured by Xia *et al.* (2019 ▸) corresponds to this model.

The scattering from a simulated structure with *x* = 1/8 is shown in the fourth column of Fig. 5 ▸. Here weak peaks of the ordered *x* = 1/6 structure remain on top of strong diffuse scattering. When *x* = 1/10 there is only diffuse scattering left, as shown in the fifth column of Fig. 5 ▸. This diffuse scattering is in agreement with the scattering measured by Xia *et al.* on Ti_0.9_NiSb and V_0.9_CoSb samples (Xia *et al.*, 2019 ▸). The calculated scattering for several other possible ground-state vacancy structures, including non-symmetry averaged planes, are shown in the Supporting information.

So far only the ground states for different compositions have been discussed. However, during the simulations it was found that it was hard to reach the ground states when starting from a random vacancy distribution unless simulated annealing was employed, or if the simulation was started from one of the ordered states close to the disordered ground state. This could suggest that real samples might also have difficulties reaching equilibrium and that they may be trapped in higher-temperature configurations if quenched. Therefore, Monte Carlo simulations were carried out to simulate the effect of temperature on the system.

**Figure 6:**
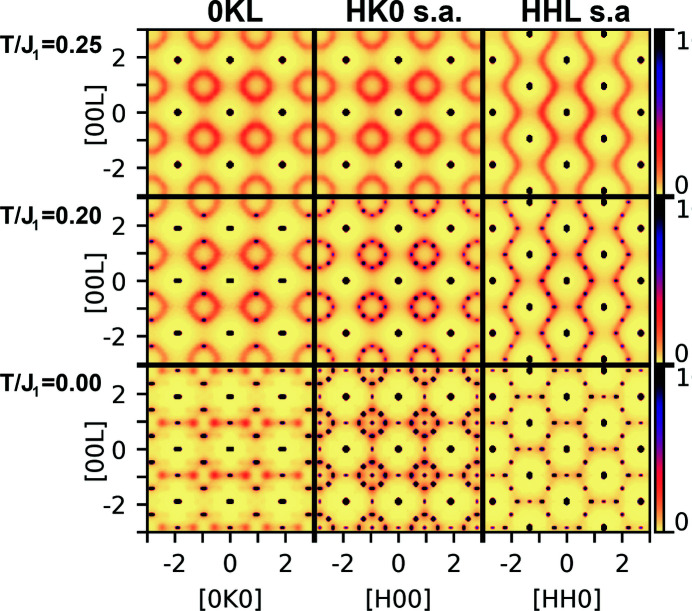
Effect of temperature on the scattering from the defective half-Heusler systems, shown here for *x* = 1/5. The left column shows the 0*KL* plane, the middle column shows the cubic symmetry averaged *HK*0 plane and the right columns show the symmetry averaged *HHL* plane at simulated temperatures *T*/*J*_1_ = 0 (bottom row), *T*/*J*_1_ = 0.2 (middle row) and *T*/*J*_1_ = 0.25 (upper row). At increased temperatures the system loses its long-range order but retains short-range order. The simulated temperature *T* is not the physical temperature but the parameter in the Monte Carlo simulation which regulates how often positive-energy moves are accepted. The same high-temperature short-range order scattering is also obtained when the stoichiometry is different, as shown in the Supporting information.

To illustrate the effect of temperature on the system, the scattering calculated for different temperatures of a Monte Carlo simulation for *x* = 1/5 is shown in Fig. 6 ▸. The left column shows the 0*KL* plane without the symmetry averaging, while the middle and right rows show the *HK*0 and *HHL* planes, which have been symmetry averaged to the Laue symmetry of the average cubic structure. The left column is therefore the expected signal for one of the *HK*0 planes for a single-domain sample, while the middle column shows the expected measurement in the *HK*0 plane for a sample composed of several domains with different orientations. The bottom row is the Monte Carlo ground state, and the simulated temperature is higher for the middle and upper row. The single-domain *HK*0 plane scattering of the low-temperature simulation, shown in the lower left corner of Fig. 6 ▸, is very similar to the measured scattering for the Nb_0.81_CoSb sample reported by Xia *et al.* (2019 ▸).

As the temperature of the simulation is increased, the long-range order disappears, leaving the short-range order. First, the peaks corresponding to the inclusions of the ordered *x* = 1/4 phase disappear, leaving peaks from the ordered *x* = 1/6 phase together with an increased amount of diffuse scattering, as seen in the middle row of Fig. 6 ▸. Then, at higher temperatures all long-range order peaks are lost, leaving the diffuse scattering of the short-range order state, as shown in the upper row of Fig. 6 ▸. This high-temperature short-range order state has similar scattering to the short-range order Nb_0.8_CoSb sample measured by Xia *et al.* (2019 ▸), as sketched in Figs. 1 ▸(*a*) and 1 ▸(*b*). The same high-temperature short-range order scattering is also obtained when the stoichiometry is different, as shown in the Supporting information. The *x* = 1/10 ground-state scattering, shown in Fig. 5 ▸, is very similar to the high-temperature scattering for all compositions (Fig. S1 in the Supporting information). This is because of the inherent disorder in all *x* < 1/6 systems as there are many states all obeying no nearest and next-nearest neighbor vacancies.

All the simulations shown here used the ratio *J*_2_/*J*_1_ = 0.48 for the relative energy penalty for a next-nearest neighbor compared with a nearest-neighbor vacancy pair, as this reproduces the observed scattering. The calculated diffuse scattering for other ratios is shown in Figs. S4 and S5.

## 5. Discussion   

The scattering measured by Xia *et al.* on different samples can all be explained through the simple model presented above for vacancy distribution in *X*_1−*x*_ *YZ* systems. The short-range order scattering from Ti_0.9_NiSb, and V_0.9_CoSb matched the simulations with *x* = 1/10, as shown in Fig. 5 ▸. The scattering from the sample with nominal stoichiometry Nb_0.81_CoSb [measured stoichiometry Nb_0.80_CoSb (Xia *et al.*, 2018 ▸)] matches the scattering from a single-orientation ground state with *x* = 1/5, shown in the bottom left of Fig. 6 ▸. Scattering from samples with nominal stoichiometry Nb_0.83_CoSb and Nb_0.84_CoSb [measured to Nb_0.81_CoSb and Nb_0.82_CoSb (Xia *et al.*, 2018 ▸)] match the model scattering in the region 1/4 > *x* > 1/6 with multiple domains of the same type oriented along different directions, which are equivalent in the average cubic structure, as shown in the second column of Fig. 5 ▸. The short-range order scattering from samples Nb_0.8_CoSb and Nb_0.8_Co_0.92_Ni_0.08_Sb match the high-temperature simulation for *x* = 1/5, as shown in the upper row of Fig. 6 ▸.

This shows that for Nb_1−*x*_CoSb samples in the range 1/4 > *x* > 1/6 some have the ground-state structure while others have the higher-temperature short-range ordering structures. One possible explanation for this is that some of the samples might have been trapped in the high-temperature state during synthesis. Indeed the samples are quenched directly from a melt during the used synthesis route (Yu *et al.*, 2018 ▸). This makes it possible that some samples are cooled faster than others, or even that some regions of the same sample are cooled faster than other regions, which could explain why some samples show the high-temperature state while others show the ground state. This would suggest that it could be possible to change between the short-range order state and the ground state by controlling the cooling rate of the synthesis, or through subsequent thermal treatment. Another explanation could be that when *x* becomes larger than 1/6 it becomes harder for the samples to reach the ground state as there are more vacancies in the structure interacting with each other. This could also explain why Xia *et al.* observed more diffuse scattering when going from *x* = 1/6 towards *x* = 1/5 (Xia *et al.*, 2019 ▸). Possibly both are true, such that either having larger vacancy concentrations or faster cooling rates would favor the short-range ordered state. This suggests that it could be possible to control the degree of vacancy order without changes in composition. This is important as the composition also dictates the amount of electrical carriers. Independent control of electrical carrier concentration and degree of vacancy order should allow further improvements in the thermoelectric properties of these systems.

It was also found that some of the samples showed scattering from only one orientation of the ground-state structure, while others showed scattering from multiple domains with different orientations, which are symmetry equivalent in the average half-Heusler structure. This suggests that it is possible to control the size of these micro-domains. This is also possibly affected by the cooling rate.

The diffuse scattering patterns of the short-range order states, as seen in Fig. 6 ▸ (top row), are similar to other previously studied systems such as the vacancy short-range order in transition-metal carbides with rock-salt structures, *e.g.* *M*C_1−*x*_ and *M*N_1−*x*_ with *M* = Ti, Nb, Ni, V (Billingham *et al.*, 1972 ▸; Sauvage & Parthé, 1972 ▸), the distribution of Li and Fe in rock-salt structure α-LiFeO_2_ (De Ridder *et al.*, 1977 ▸), oxygen and fluorine distribution in oxyflouride K_3_MoO_3_F_3_ (Withers *et al.*, 2003 ▸), short-range order in Mg_1−*x*_Yb_2*x*/3_□_*x*/3_S and related systems (Urones-Garrote *et al.*, 2005 ▸; Withers *et al.*, 2007 ▸), among others (Gusev, 2006 ▸). What is common for these systems is that they have substitutional disorder, either of two types of elements/ions or of one element and vacancies on an f.c.c. lattice, often a substructure of the structure. Many of these systems have been explained in terms of a cluster model where the structure is built from octahedral clusters each with as close as possible stoichiometry to the sample. This local invariance of stoichiometry, often attributed to Linus Pauling, can in many of these systems be fulfilled without long-range order. The result of the invariant octahedral clusters in an f.c.c. substructure is that the diffuse scattering is limited to the curved surface satisfying (Sauvage & Parthé, 1972 ▸, 1974 ▸, De Ridder *et al.*, 1976 ▸)
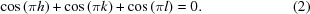
The intersection of this surface with two planes is identical to the drawn lines in Figs. 1 ▸(*a*) and 1 ▸(*b*). If the octahedral cluster model is also used to explain the scattering observed for the short-range order in the half-Heusler systems, the octahedra in the f.c.c. substructure should be as close as possible to the average stoichiometry. An example of one such octahedra is the six side-centered atoms of the f.c.c. unit cell. However, every site of the f.c.c. substructure is contained in six octahedra. Those six octahedra are made from all nearest and next-nearest neighbors to the site. For *x* ≤ 1/6, this model would imply that all octahedra have at most one vacancy. This is equivalent to all vacancies having no nearest or next-nearest neighbors, as is the case for the ground states found in this study. For 1/4 > *x* > 1/6, each octahedra would sometimes contain two vacancies in the cluster model. Although this is also true for the ground states found here, the two are not equivalent, as the cluster model will allow some nearest neighbors, while the found ground states do not have nearest-neighbor vacancies, only some next-nearest neighbors in this range. However, the high-temperature states found here for 1/4 > *x* > 1/6, for which the scattering is shown in Fig. 6 ▸ (top row), are equivalent to the octahedral cluster model.

Some previously reported systems also show similar types of ordering as the half-Heusler samples. De Ridder *et al.* observed that the alloy Ni_4_Mo changes structure when annealed (De Ridder *et al.*, 1976 ▸). One of the obtained phases shows an electron-diffraction pattern which matches the one calculated here for the *x* = 1/5 structure suggested by Zeier *et al.* (2017 ▸) shown in Fig. 5 ▸ (right column). They also found that after annealing a sample of Ni_3_Mo a diffraction pattern with the 8-point ring was obtained, similar to the ones shown here. These ordered systems can also be explained in terms of the octahedral cluster model (De Ridder *et al.*, 1976 ▸; Sauvage & Parthé, 1974 ▸) with the addition that certain configurations of the octahedra are more favorable that others, for example that vacancies on the same octahedra tend to be opposite. This is equivalent to vacancies not having nearest neighbors, making the model equivalent to the ones found in this study.

The short-range order observed in these half-Heusler systems therefore belongs to a more general type of short-range order found in many systems with substitutional or vacancy disorder in f.c.c. substructures. Although these systems are chemically very different, the rule that local stoichiometry should be conserved leads to the same topology of the diffuse scattering. Indeed there are many such families of equivalent short-range order found in chemically very diverse systems (Keen & Goodwin, 2015 ▸). The link between local rules and the topology of diffuse scattering is very interesting. The approach of using the invariant cluster models was already developing in 1974 (Sauvage & Parthé, 1974 ▸). A general approach to extinction rules in diffuse scattering based on symmetry was developed by Withers *et al.* (2010 ▸). The diffuse scattering for a large number of networks made from invariant building blocks obeying simple rules was shown by Overy *et al.* (2016 ▸). Recently, a new way of treating diffuse scattering has emerged, describing the structures using a modulation-wave approach (Withers, 2015 ▸). This method lends itself very well to describing types of systems where diffuse scattering is limited to specific surfaces, such as the case found here. Another very promising approach is to describe the diffuse scattering using a disordered superspace model for the structure, which allows one to describe seemingly very complex systems using only a few parameters (Schmidt & Neder, 2019 ▸).

## 6. Conclusions   

Defective half-Heusler systems *X*_1−*x*_ *YZ* with large amounts of vacancies have previously been found to show interesting scattering patterns, indicating short-range order in some samples and long-range order in others. Here, we have shown that all observed scattering patterns can be explained in terms of a simple model. The model assumes that vacancies tend to avoid being close to each other. The vacancies are distributed on a face-centered cubic substructure of the half-Heusler structure. By avoiding nearest-neighbor vacancy pairs and minimizing next-nearest neighbor vacancy pairs, the observed ordered phases can be explained. Furthermore, by simulating the effect of temperature on the system, the observed short-range order phases can be explained as quenched non-ground-state phases. It is also found that some of the reported measurements come from a single orientation of the ground-state phase, while other reported measurements originate from several ground-state domains with different orientations.

As the observed short-range order state can be explained through this model as a quenched high-temperature state, while the observed ordered phase is the ground state of the same system, it suggests that the occurrence of the two phases can be controlled through thermal treatment of samples. Such control of the degree of disorder/order in the system should in turn allow tunable thermoelectric properties, as the thermal conductivity is expected to depend on the degree of long-range order. It has previously been observed that changes in sample composition can affect whether short- or long-range vacancy ordering is obtained. However, the composition also dictates the number of electrical carriers. This study therefore suggests that the degree of vacancy order, and thereby the thermal conductivity, can be controlled independently of carrier concentration using thermal treatment, allowing further optimization of thermoelectric properties.

## Supplementary Material

Supporting information. DOI: 10.1107/S2052252520005977/fc5045sup1.pdf
