# Supplementary material for: A simple model for vacancy order and disorder in defective half-Heusler systems
Source: IUCrJ. 2020 Jun 6;7(Pt 4):673–80. doi: 10.1107/S2052252520005977 (PMC7340261; doi:10.1107/S2052252520005977)
Supplement: Supplementary file 1 [file m-07-00673-sup1.pdf]

IUCrJ

Volume 7 (2020)

Supporting information for article:

A simple model for vacancy order and disorder in defective half-Heusler systems

Nikolaj Roth, Tiejun Zhu and Bo B. Iversen

S1. High-temperature simulations

In the figure below the calculated scattering for high-temperature (T/J_1=0.3) simulations is shown for several vacancy concentrations, x in $X_{1-x}YZ$. The top row shows the HK0 plane, while the bottom row shows the HHL plane. All compositions show similar diffuse scattering. For lower vacancy concentrations the diffuse signal becomes weaker.

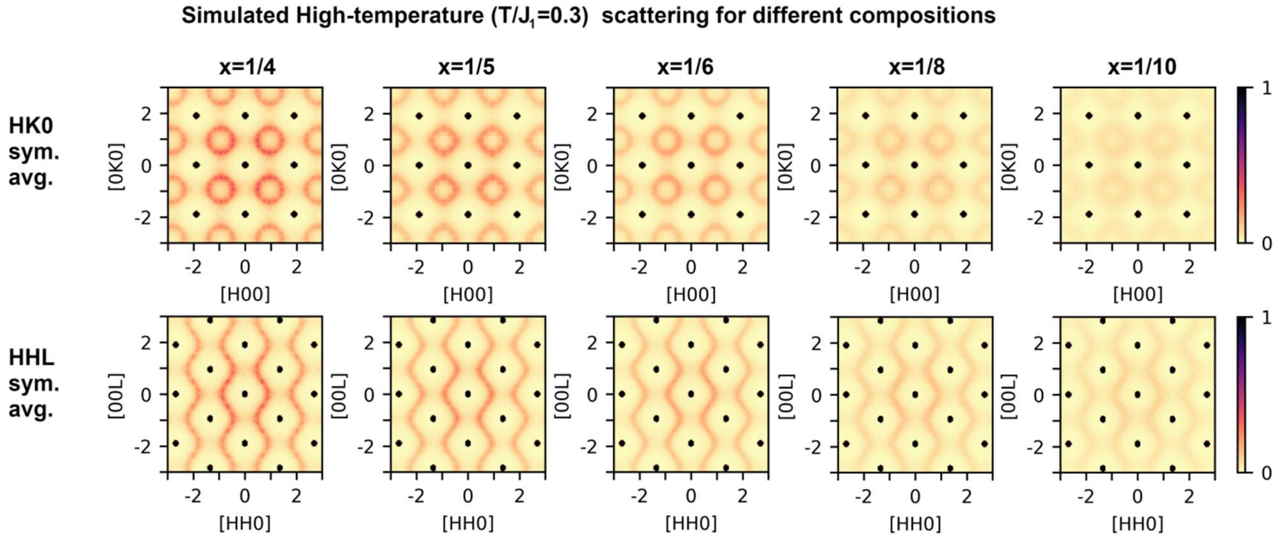

Figure S1 Simulated high-temperature scattering.

S2. Other vacancy structures

For x=1/6 there are infinitely many possible configurations of vacancies which satisfy no nearest or next-nearest neighbors. These are shown on the next page (figure S2). In the configuration marked A1 every row of the blue triangles is identical, while in A2 the columns of blue triangles are shifted in their y-coordinate, making every second column identical. An infinite number of configurations between these are possible where each column is either identical or shifted with respect to the previous. An example of this is shown in the configuration marked AD (disordered). Red lines are used to visualize the shift in columns. Another ordered configuration is shown marked B1. This configuration has every second row of blue triangles identical. If every row was identical it would be the same structure as A2 (with change of colors and a 90 degree rotation). Similarly disordered configurations between these cases still fulfill the same rules. An example is shown marked BD (disordered). More types of ordered configurations are marked C1 and C2. The difference between them is whether each row is shifted right or left with respect to the previous row. Similarly disordered configurations between these cases still fulfill the same rules. An example is shown marked CD (disordered). Calculated scattering for these models are shown in figure S3.

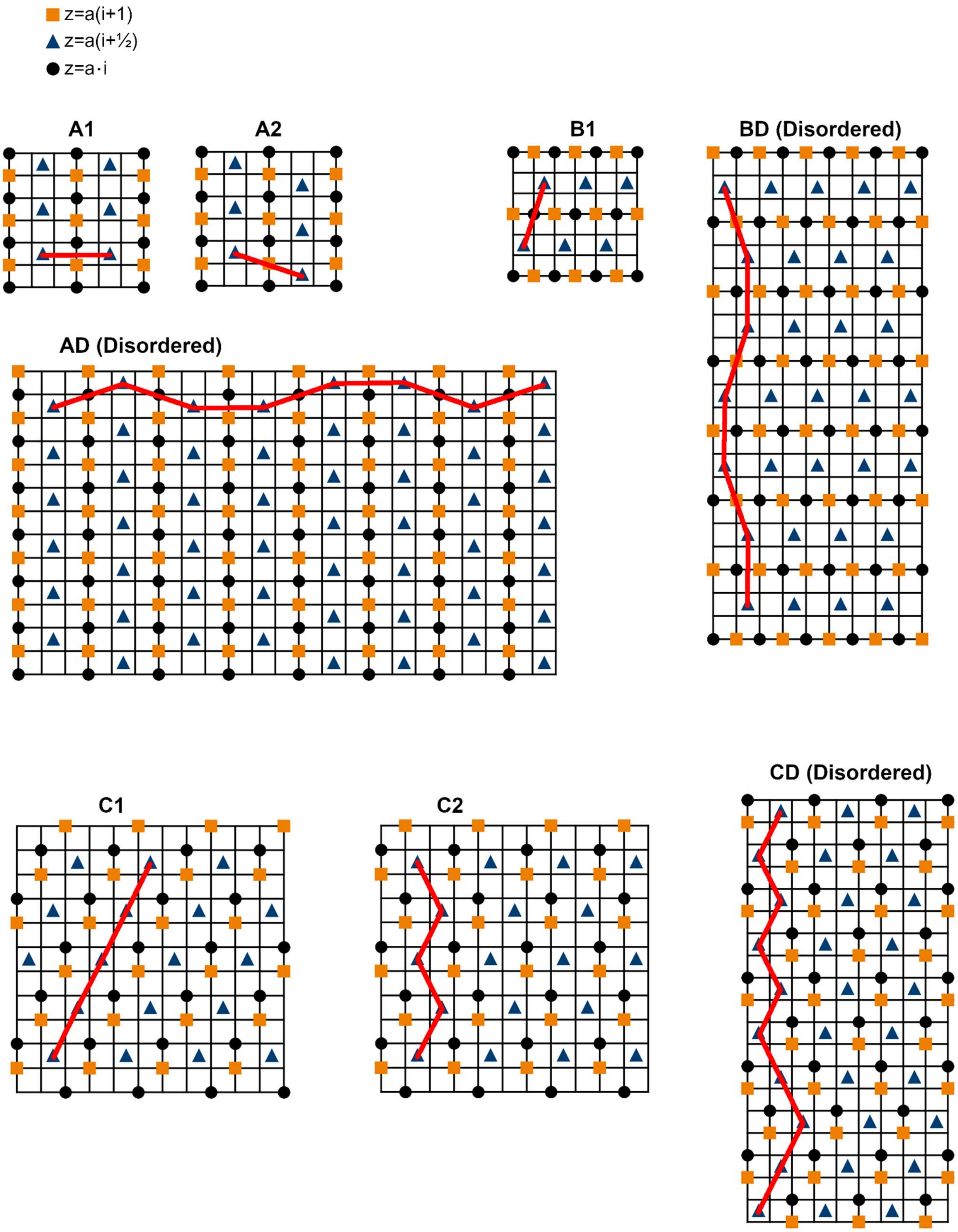

Figure S2 x=1/6 structures with no nearest or next-nearest neighbors.

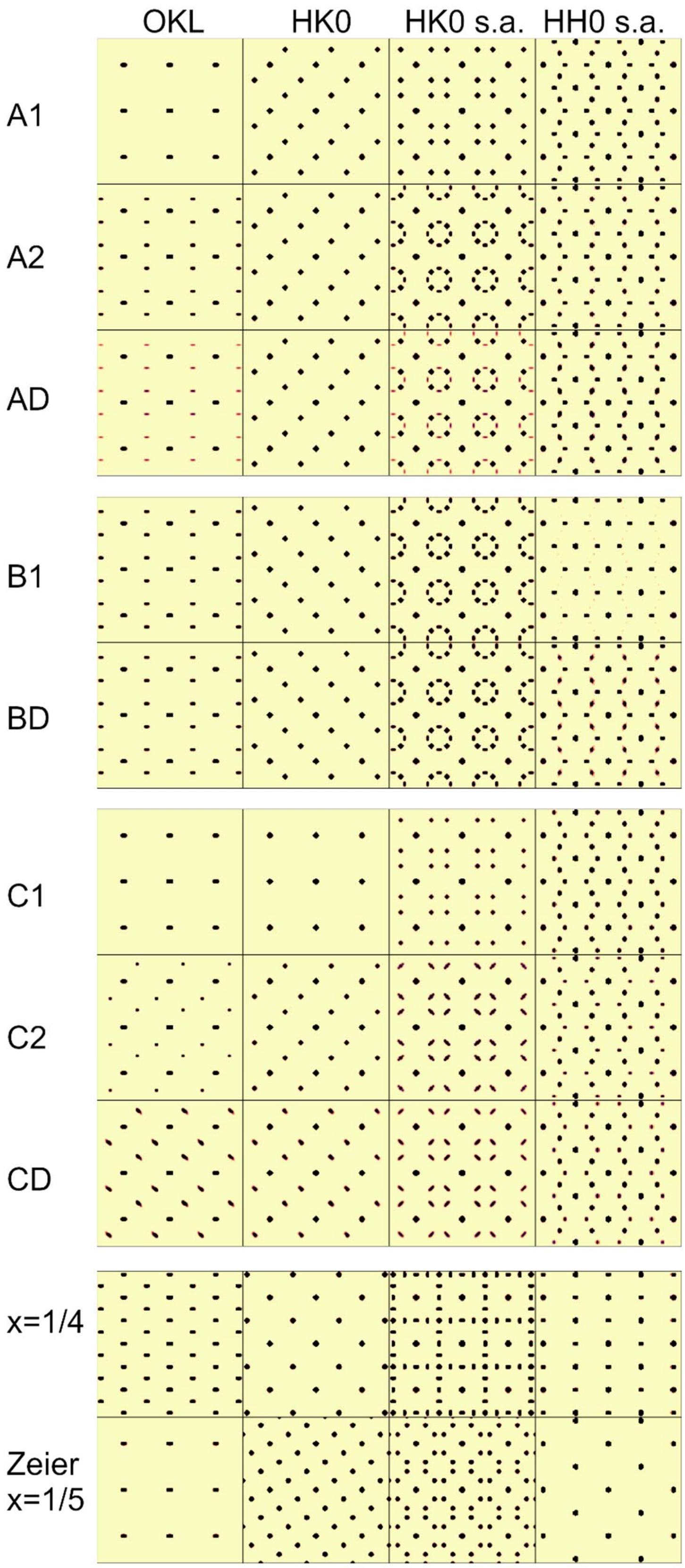

Figure S3 Calculated scattering for several ground-state vacancy structure models.

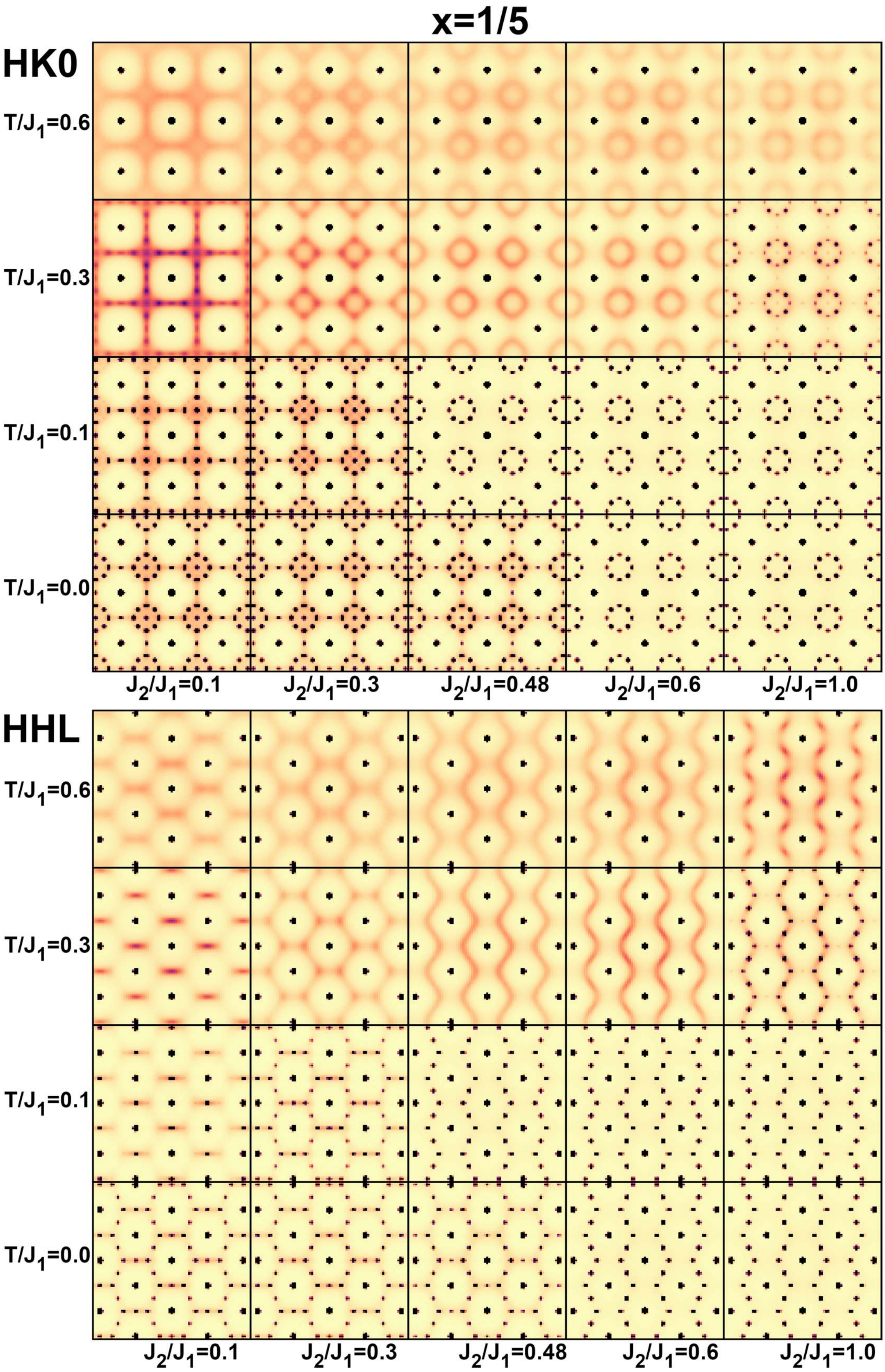

Figure S4 Calculated scattering for different interactions and temperatures with x = 1/5.

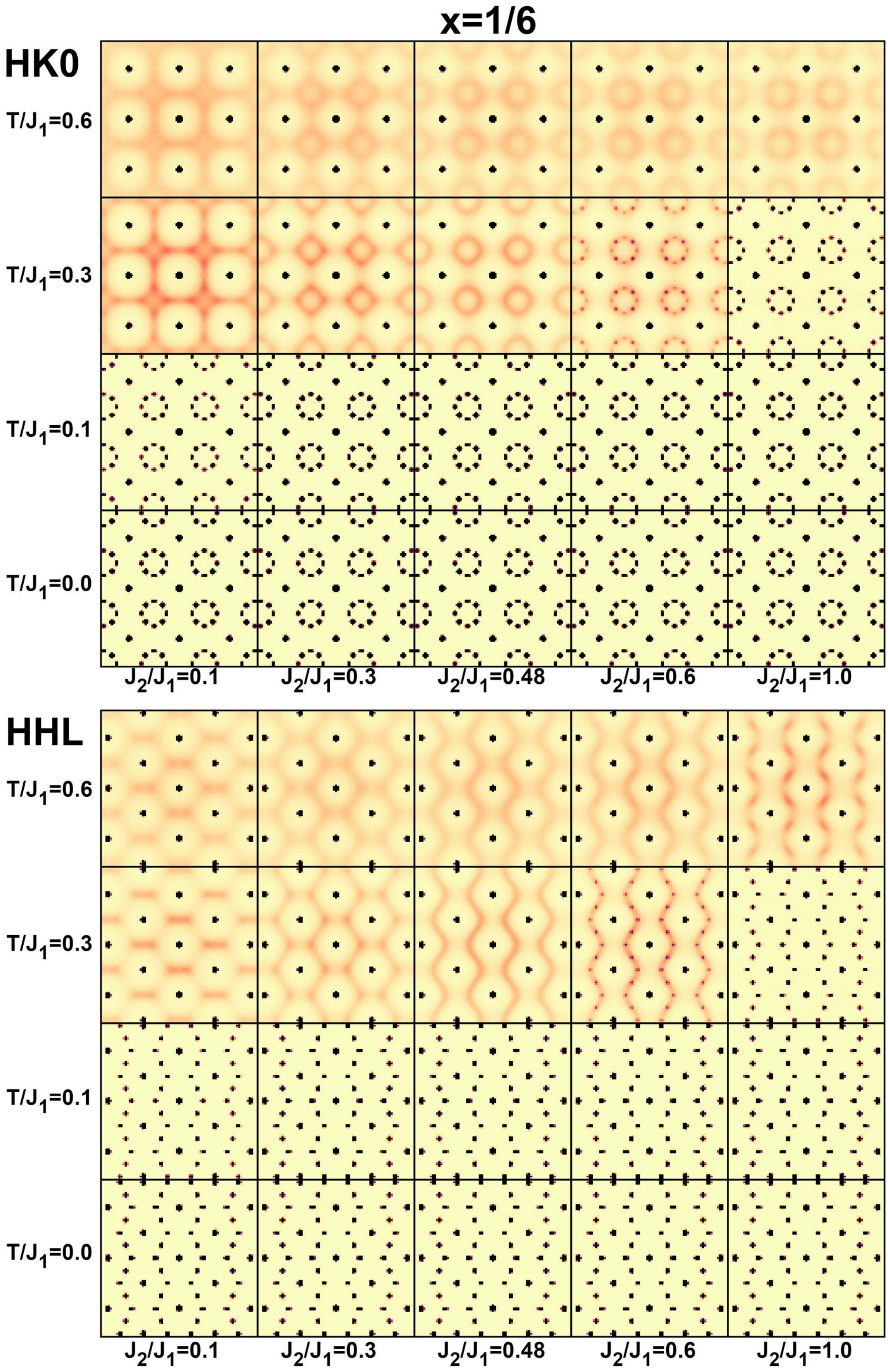

Figure S5 Calculated scattering for different interactions and temperatures with x = 1/6.
